# Interconnectivity among different nonsuicidal self-injurious methods – a network analysis

**DOI:** 10.1186/s12888-025-07045-2

**Published:** 2025-07-01

**Authors:** Melinda Reinhardt, Kenneth G. Rice, Hunggu Cho, Zsolt Horváth

**Affiliations:** 1 Institute of Psychology, ELTE Eötvös Loránd University, Budapest, Hungary; 2 14th District Medical Center, Child and Adolescent Psychiatry, Budapest, Hungary; 3 Matheny Center for the Study of Stress, Trauma, and Resilience, Department of Counseling and Psychological Services, Georgia State University, Atlanta, USA

**Keywords:** Nonsuicidal self-injury methods, Network analysis, Community adults

## Abstract

**Background:**

An important indicator of self-harm severity is the co-occurrence of different nonsuicidal self-injurious (NSSI) behaviors. However, there is little research on how different self-injurious behaviors (e.g., cutting, biting, burning, carving) are related. In contrast to person-centred approaches, variable-centred network analysis helps to identify the most meaningful associations between different forms of NSSI behavior, thus allowing the identification of structural patterns in different NSSI methods. Based on network analysis, it will be possible to examine which NSSI methods are the most central and which are most closely linked to other methods.

**Methods:**

We used network analysis to investigate interconnections between 12 different self-harm methods. The Inventory of Statements About Self-Injury [24] was utilized to assess the types and frequency of NSSI in a community adult sample.

**Results:**

More than one-third (39.7%; *n* = 744) of 1873 adults reported at least one episode of NSSI in their lifetime. Most (77%) of those engaged in NSSI used more than one NSSI method. The most frequently used NSSI methods were hitting self, interfering with wound healing, pinching, biting, and severe scratching. In the NSSI methods network analysis, severe scratching, cutting, and pinching had the highest rates of interrelationship with other NSSI behaviors. Moreover, the interconnectedness of certain specific NSSI-methods (i.e., cutting with burning and carving; severe scratching with pinching, biting and hitting self) have a greater risk of co-occurrence (versatility).

**Conclusions:**

Although different NSSI methods occurred as distinct entities, some play a more central role in the network. Our results suggest that the NSSI methods identified as central should be given more attention in clinical settings as these behaviors may indicate the severity of the condition. Specifically, when assessing and treating those who engage in self-harm, clinicians may find it useful to create a detailed map of the person-specific NSSI-methods to inform risk assessment and treatment.

**Supplementary Information:**

The online version contains supplementary material available at 10.1186/s12888-025-07045-2.

According to a growing number of self-harm studies, one of the most important indicators of the severity of nonsuicidal self-injury (NSSI) is the combination of different self-injurious methods, referred to as “versatility” [3]. The use of multiple NSSI methods is significantly connected to higher clinical risk, especially suicidal self-injury (SSI; Nock et al. [33]. However, there is still a gap in the literature regarding how the different NSSI methods relate to each other. Although the interrelationship of NSSI behaviors with model-based methods for clustering (e.g., Latent Class Analysis; LCA, Latent Profile Analysis; LPA) has been explored, we are unaware of any study that has used network analysis to examine the connectedness of various NSSI methods. Therefore, the present study uses network analysis to explore the interconnectivity of 12 NSSI methods most frequently reported in the literature. Network analysis based on graph theory can help us to better understand which NSSI methods are most closely linked to others, which are the most central, and to visualize the interrelationship between different NSSI methods. The general research aim is to determine whether the different NSSI behaviors are linked in any way.

NSSI is an umbrella term that covers various forms of behavior that hurt one’s own body intentionally but without suicidal intent [25]. In terms of its mechanism, NSSI can be defined as the behavioral imprint of maladaptive coping strategies that manifest on the body surface [21]. Commonly reported examples of NSSI behaviors include cutting, biting, burning, carving, pinching, severe scratching, banging or hitting self, pulling hair, interfering with wound healing, rubbing skin against rough surface and sticking self with needles can be included [24]. Rare and specific forms are also known, such as touching the tongue to a cold object, choking, deliberately chewing the inside of the mouth, breaking bones, mutilating genitals or swallowing dangerous substances [19, 50]. Research shows consistency in that cutting, scratching, hitting or banging, and burning are the most frequently used forms of NSSI [25]. In a general USA adult sample, cutting, carving, scratching, picking, biting, and burning the skin, as well as hitting self, and interfering with wound healing were the reported NSSI methods [22]. However, there are gender differences in engaging in these methods [5]. Males are more likely to hit themselves, whereas females tend to engage in methods that are more likely to cause bleeding, such as scratching, cutting, and carving the skin [7, 43].

In community populations, the prevalence of NSSI has shown an increasing trend over the past decade, mainly among adolescents [11] and young adults [47]. In these life stages, up to 50% lifetime prevalence of at least one NSSI episode has been reported [9]. Nonetheless, in the lifetime history of NSSI, a relevant decreasing trajectory can be detected for adulthood in the general population. In a recent nationally representative survey in England, the lifetime prevalence of NSSI nearly halved among people over 30 [30].

With regard to NSSI, it is very important to consider its severity. Several different indicators can express the seriousness of self-harm. One (traditional) index of severity is the frequency of NSSI acts. There is no consensus in the literature on how many NSSI acts can be considered occasional and how many NSSI acts can be considered repetitive and chronic self-injury. However, previous research suggests that repetitive, especially chronic, self-injury is associated with clinical severity (e.g., higher psychiatric symptoms or suicidality risk; Lloyd-Richardson et al. [27]. Another indicator of severity is whether the self-injurious act results in a physical injury that requires medical attention [30]. We can also consider the seriousness of NSSI according to the method used by the person. There are low-severity forms (e.g., hitting and preventing wounds from healing) and moderate or high-severity forms of NSSI (e.g., choking or cutting; Lloyd-Richardson et al. [27]. However, in many cases, categorization is problematic because several methods can lead to minor but more serious injuries, even requiring medical attention [41].

Recent research increasingly suggests that the number of applied NSSI methods (versatility) is a fundamental marker of the severity of self-harm. This indicator is particularly important because in a significant proportion of cases, both in clinical (e.g., Reinhardt et al. [37] and community samples (e.g., Klonsky, [22] participants with NSSI history reported engaging in multiple methods. Engaging in various methods of NSSI is associated with severe intrapersonal and interpersonal mental health symptoms [50], and suicide [33, 44]. Engagement in multiple NSSI methods has been linked to earlier onset of NSSI, more frequent NSSI, longer persistence of NSSI, and higher risk of suicide [2]. Based on the results of a study involving more than 2,700 emerging adults, it can be suggested that a more reliable indicator of NSSI severity is the combination of its frequency and versatility [3]. There is also evidence that versatility has an impact on the link between NSSI frequency and clinical severity [4]. These empirical results provide support for the Integrated Model of self-injurious behaviors (SIB), which suggests that engaging in diversified methods of NSSI might moderate the relationships between NSSI and the acquired capability for suicide, which in turn can result in suicidal behavior [17]. Based on empirical evidence, the model also assumes that the type of NSSI behavior is also an important factor in the risk of suicidal behaviors. More serious forms of NSSI (e.g., cutting), compared to low-severity methods of NSSI (e.g., hair pulling), can boost the probability of engaging in suicidal acts [17]. However, among youth who received psychiatric treatment, low-severity methods of NSSI (hitting or banging and preventing wound healing) were related to suicidal ideation, whereas more severe methods (e.g., burning or swallowing dangerous substances) were not [45]. The same study also confirmed that the use of multiple methods of NSSI was associated with more frequent suicidal ideation. Thus, evaluating the heterogeneity of NSSI methods, rather than the methods or their severity, may help clarify the association between NSSI and suicidal ideations.

Based on the importance of exploring the heterogeneity of NSSI methods, it is worth paying attention to whether any links between the various methods of self-harm can be identified, and whether they are connected to each other in any context. This has so far been explored in the literature by person-oriented approaches (e.g., LCA, LPA). These techniques have helped to highlight that persons who engage in NSSI are not a homogeneous class, but rather subgroups can be distinguished according to their NSSI characteristics, such as NSSI functions, NSSI frequency, or NSSI methods. The majority of studies, both among adults and adolescents, point in the same direction by showing clear heterogeneity in NSSI, and usually 3–5 subgroups with similar content emerge. The severe NSSI subgroup can be featured with a high frequency of NSSI and multiple NSSI endorsements associated with higher psychological distress and more severe mental illness symptoms [23]. Similarly, a moderate group of NSSI consistently stands out with moderate NSSI endorsement in various NSSI methods [10]. In one study, the group with the highest level of NSSI-versatility had the highest vulnerability for psychopathology [6]. A similar picture emerges among adolescents, where the NSSI-versatility class had the most psychological vulnerability and poorer mental health [38, 42]. One adolescent study also showed that in the severe NSSI group, the majority of the numerous NSSI methods used (e.g., cutting, sticking self with needles, biting) can result in severe body tissue damage, while in the low-risk group for NSSI low severity forms of NSSI (predominantly hitting and interfering with wound healing) occurred with low frequency [38].

Although past research with person-centered methods attempted to explain the importance of the number of NSSI methods and their relationship with mental health, the relationship between the NSSI methods has not been explored sufficiently. Network analysis provides an approach to address that limitation. Although there exist a limited number of network analyses of the relationship between NSSI and the suicidal spectrum [51] as well as psychological (e.g., depressive symptoms, internalizing problems, risk behaviors; Mancinelli et al. [31] and psychosocial (e.g., childhood trauma, social support; Zhou et al. [51] factors, or between NSSI and aggressive acts [40], the exploration of the interconnectivity in the NSSI methods system is clearly a research gap. Thus, to fill this gap, the present study used network analysis to investigate the connections between numerous NSSI methods. Network analysis is an exploratory approach that helps to understand the structure of systems (in this case, the structure of NSSI methods) by pointing out relations (“edges”) between variables (“nodes”) and the strength of these ties [13]. While person-centered approaches (e.g., LCA, LPA) allow for the identification of group-level co-occurrence patterns of NSSI, network analysis offers a broader perspective on the interrelationships between different NSSI behaviors. Specifically, it enables the identification of the most meaningful links between various NSSI methods and helps determine which forms of NSSI may be more central or more peripheral within the network (i.e., exhibiting more and stronger vs. fewer and weaker interrelationships, respectively). Moreover, similar to other variable-centered techniques (e.g., confirmatory and exploratory factor analysis), network analysis can reveal potential clustering and structural patterns within NSSI behaviors. A distinctive advantage of network analysis is its ability to capture relationships at the level of individual NSSI behaviors rather than focusing on latent constructs or predefined categories. By mapping direct and indirect associations between individual behaviors, network analysis highlights connections that may otherwise remain obscured in aggregate-level analyses.

## Current study

This study aims to explore the relationships between different NSSI methods and the strengths of the associations using a network analysis approach. Therefore, the interrelations between 12 NSSI methods (cutting, biting, burning, carving, pinching, pulling hair, severe scratching, banging or hitting self, interfering with wound healing, rubbing skin against rough surface, sticking self with needles, and swallowing dangerous substances) were investigated. The main objective was to identify which NSSI behaviors have important or central connections with other behaviors in the network, as well as those behaviors that are more marginal in the network. We also sought to determine the possible overlaps between these apparently diverse NSSI types. In these overlapping areas within the network, NSSI methods that are more closely related are assumed.

According to the aforementioned results, which recognized NSSI versatility as a main indicator of NSSI severity, we expected that the different NSSI methods would be relatively distinct yet interrelated entities, with certain NSSI methods would likely playing a more central role in their interrelationship with other methods (i.e., as indicated by higher number and stronger associations). It was hypothesized that the more serious forms of NSSI would exhibit stronger and denser relationships with other modes. The results have the potential to further refine the picture of how various NSSI methods are linked. More specifically, network analysis could reveal how NSSI methods may interact, which could improve targeting important intervention points in high-risk severity groups for NSSI.

## Method

### Participants and procedure

A total of 3,045 Hungarian community adults started a larger online survey, and 1,873 completed the questionnaire package. For purpose of this study, only the NSSI results from that survey were used. 1,172 incomplete responses were excluded from the analysis as these respondents stopped completing the questionnaire package. Of the total respondents (*N* = 1,873), 744 (39.7%) had been engaged in NSSI at least once in their life. The analyses were, by definition, limited to this group. Therefore, the descriptive data for this NSSI sample are given below. Just over two-thirds (*N* = 573; 77.0%) of the NSSI group were female, and 171 (23%) were male. At this point, it is particularly important to note that we cannot control for possible gender differences in the analyses due to the inbalanced gender ratio. However, the imbalanced gender ratio limits the generalizability of our results. Research suggests while there are no gender differences in the frequency of NSSI, and the number and variety of methods used (e.g., Victor et al. [46], however differences may exist in engagement in certain NSSI methods. Women are more likely to engage in cutting, while men are more likely to engage in hitting or banging themselves [7, 43]. These gender differences and the significant predominance of female respondents in the sample may have implications for the network structure of the NSSI methods explored.

Ages ranged from 18 to 74 years, and the average age of the NSSI respondents was 29.87 (SD = 11.01). Most of the NSSI sample fell into the young adult age range (18–39 years; *N* = 595; 80.0%), with smaller proportions of middle-aged adults (40–59 years) and senior adults (> 60 years, *N* = 147; 19.7%); two participants (0.3%) did not specify their age. The overwhelming majority of the NSSI sample lived in the capital (*N* = 361; 48.5%) or other cities (*N* = 253; 34.0%) and graduated from university (*N* = 395; 53.1%) or high school (*N* = 321; 43.1%). Most of those who engaged in NSSI (*N* = 512; 68.8%; 27.33% of the total sample) did so more than a month ago (NSSI in the past), while just under a third (*N* = 232; 31.2%; 12.38% of the total respondents) did so in the past month (current NSSI). Most (*N* = 573; 77%) engaged in more than one method; on average, 3.66 methods were reported (SD = 2.28; min = 1, max = 12).

The data collection period was from the beginning of March 2019 to the end of July 2021. A non-probability convenience sampling method was used in the community recruitment. The research was advertised on various social media online platforms and in a Hungarian journal on psychological topics for lay readers. Data were collected using Qualtrics, a web-based survey platform. Participants voluntarily provided informed consent and participated anonymously in the research.

The study was approved by the Institutional Review Board of the Eötvös Loránd University Faculty of Education and Psychology, and the work was conducted in accordance with the Declaration of Helsinki [48].

### Measure

Lifetime frequency of 12 different self-injurious methods (i.e., cutting, biting, burning, carving, pinching, pulling hair, severe scratching, banging or hitting self, interfering with wound healing, rubbing skin against rough surface, sticking self with needles, and swallowing dangerous substances) was measured with the Hungarian version [36] of the Section I of the Inventory of Statements about Self-Injury (ISAS; Klonsky & Glenn, [24]. The first section of the questionnaire measures the number of intentional engagements in different self-injurious methods in a lifetime. However, considering the statistical characteristics of the data as well as the recommendations of previous studies examining the frequency of NSSI, the original variables measuring the number of engagements in each NSSI method were not used. Instead, these variables were recoded into ordinal variables with three categories. Following the categorization suggested by Gratz et al. [16]: (i) 0 lifetime NSSI episodes were considered an absence of self-harm, (ii) 1–9 lifetime NSSI episodes were considered occasional self-harm, and (iii) 10 or more NSSI episodes were described as recurrent self-harm. Overall, while the primary rationale for the applied transformation was to mitigate statistical issues and enhance the robustness of the analyses, it should also be acknowledged that this approach reduced variability and may have led to some loss of information regarding the NSSI variables. The recoding of these variables was justified by their extremely skewed, non-normal distribution, along with the presence of outliers and very wide range, which could have biased their consideration as continuous variables. Furthermore, the assumption of equality between the mean and variance, required for a Poisson-type distribution, was not met (see Supplementary Table 1). Thus, the transformation of the original NSSI variables into ordinal measures was statistically justified.

### Data analysis

As preliminary analyses, the lifetime frequencies of each NSSI behavior were calculated, along with bivariate Spearman correlations between different NSSI behaviors.

Network analysis was performed to estimate and interpret a network of NSSI behaviors using the bootnet package in R [14]. Supplementary materials contain the codes for the network analyses. A weighted and undirected network was estimated using the mixed graphical models (mgm) approach [18]. The mgm approach extends the traditional Gaussian graphical model [29] by allowing for the estimation of networks with various types of variables, including continuous, ordinal, and count variables. This method was considered suitable for the present network analysis as it included 12 nodes representing ordinal variables that measured the lifetime frequency of each NSSI behavior (with 3 categories: never, 1–9 times, 10 or more times). Therefore, 66 edges were estimated in the network. Edge weights were estimated to represent conditional associations between variables, based on pairwise interactions from log-linear models. That is, a non-zero and depicted edge between two nodes in a network indicated that there is a relationship between the given two variables after controlling for the effects of the other NSSI behaviors.

In order to obtain a sparse and parsimonious network and to control for the false positive associations in the network, edge selection followed the AND rule, and model selection was guided by the Extended Bayesian Information Criterion (EBIC). The use of the AND rule ensured that an edge was included in the final network only if it was nonzero in both directions between two given variables (e.g., from variable A to variable B as well as from variable B to variable A). Based on previous recommendations, the value of the EBIC hyperparameter was set at 0.5 [12, 14, 15]. A higher hyperparameter allows the regularization to put more emphasis on simplicity (parsimony). This method helps highlight only meaningful and substantial associations in a network while setting small associations to zero. Additionally, model estimation was performed using 20-fold cross-validation to enhance the robustness of the estimated edges.

The next step of the analyses aimed to identify which NSSI behaviors have important and central roles in the network. For this purpose, the standardized centrality index of strength was primarily considered. The strength for a given node is calculated by summing absolute edge weights related to all other nodes in the network. Thus, it can quantify the relationship of a given node on the other nodes in the network. That is, a variable with high strength can be interpreted as having numerous or strong associations with other variables in the network, indicating a more central role and a greater degree of interconnectedness. In contrast, low strength suggests that a variable has fewer or weaker associations, implying a more peripheral and isolated role within the network [12, 14, 15, 28]. The present study restricted centrality analysis to the strength index, as previous research has consistently shown it to be more stable and replicable than closeness and betweenness, which are prone to greater instability and have limited interpretability and practical utility in psychological networks [8, 14, 20]. Furthermore, the results for the expected influence centrality index were not reported, as all edge weights in the final network model were positive, making it equivalent to the strength centrality index. As a supplementary analysis, Zhang’s clustering coefficient (Zhang & Horvath, 2005) was also computed. High levels of Zhang’s clustering coefficient can show a redundancy of a given node by considering the connectedness of a given node’s neighbors. That is, a high Zhang’s clustering coefficient may indicate that the given variable has less unique explanatory value, as it exhibits considerable local connectivity and overlapping associations with related variables. In contrast, a low Zhang’s clustering coefficient suggests that the variable is weakly connected or independent from its neighbors, implying a more distinctive and independently functioning role [12].

Finally, the stability and accuracy of the network parameters were examined. Three main methods were based on Epskamp et al. [14]. First, the accuracy of each edge weight was tested by calculating confidence intervals (CIs) for edge weights based on a non-parametric bootstrap with 1000 samples. This analysis can help investigate the accuracy of the strength and direction of associations in a network. Second, a case-dropping subset bootstrap analysis was performed with 1000 samples. This method aims to explore the stability of different centrality indices. Namely, network analysis was re-performed on subsets of the data by dropping increasing proportions of the participants, and correlation estimates were calculated between the original and case-dropping network analyses regarding centrality estimates. Thus, the value of the correlation stability (CS) coefficient indicates the maximum proportion of cases that can be dropped (with 95% probability) to have at least 0.70 (i.e., very large) correlation between the centrality indices from the original dataset and the subsets of data. Values of the CS coefficient ≥ 0.50 indicate optimal stability, whereas values from 0.25 to 0.49 represent adequate stability of the centrality measures. Third, bootstrapped difference tests with CIs were computed using 1000 samples to investigate significant differences between edge weights as well as node-based strength centrality values [14].

## Results

### Descriptive statistics

Table 1 summarizes lifetime frequencies and abbreviations of NSSI behaviors. Supplementary Table 2 presents the bivariate Spearman correlation estimates between frequencies of the 12 NSSI behaviors.

**Table 1:** Lifetime frequencies of different nonsuicidal self-injury (NSSI) behaviors

Abbreviation	NSSI behavior	Frequency		
		Never *N* (%)	1–9 times *N* (%)	10 or more times *N* (%)
CUT	Cutting	492 (68.52%)	126 (17.55%)	100 (13.93%)
BIT	Biting	395 (55.40%)	142 (19.92%)	176 (24.68%)
BRN	Burning	621 (86.73%)	75 (10.47%)	20 (2.79%)
CRV	Carving	535 (74.83%)	124 (17.34%)	56 (7.83%)
PIN	Pinching	359 (50.42%)	147 (20.65%)	206 (28.93%)
PUL	Pulling hair	559 (78.62%)	82 (11.53%)	70 (9.85%)
SCR	Severe scratching	434 (60.70%)	146 (20.42%)	135 (18.88%)
BNG	Banging or hitting self	325 (45.84%)	183 (25.81%)	201 (28.35%)
WND	Interfering with wound healing	341 (48.23%)	92 (13.01%)	274 (38.76%)
RUB	Rubbing skin against rough surface	620 (86.96%)	46 (6.45%)	47 (6.59%)
STC	Sticking self with needles	610 (85.67%)	54 (7.58%)	48 (6.74%)
SWL	Swallowing dangerous substances	671 (94.91%)	26 (3.68%)	10 (1.41%)

*Note. *Missing cases (*N* = 26–37) in each variable are not considered for the proportion statistics (*%*).

### Network of NSSI behaviors

**Fig. 1:**
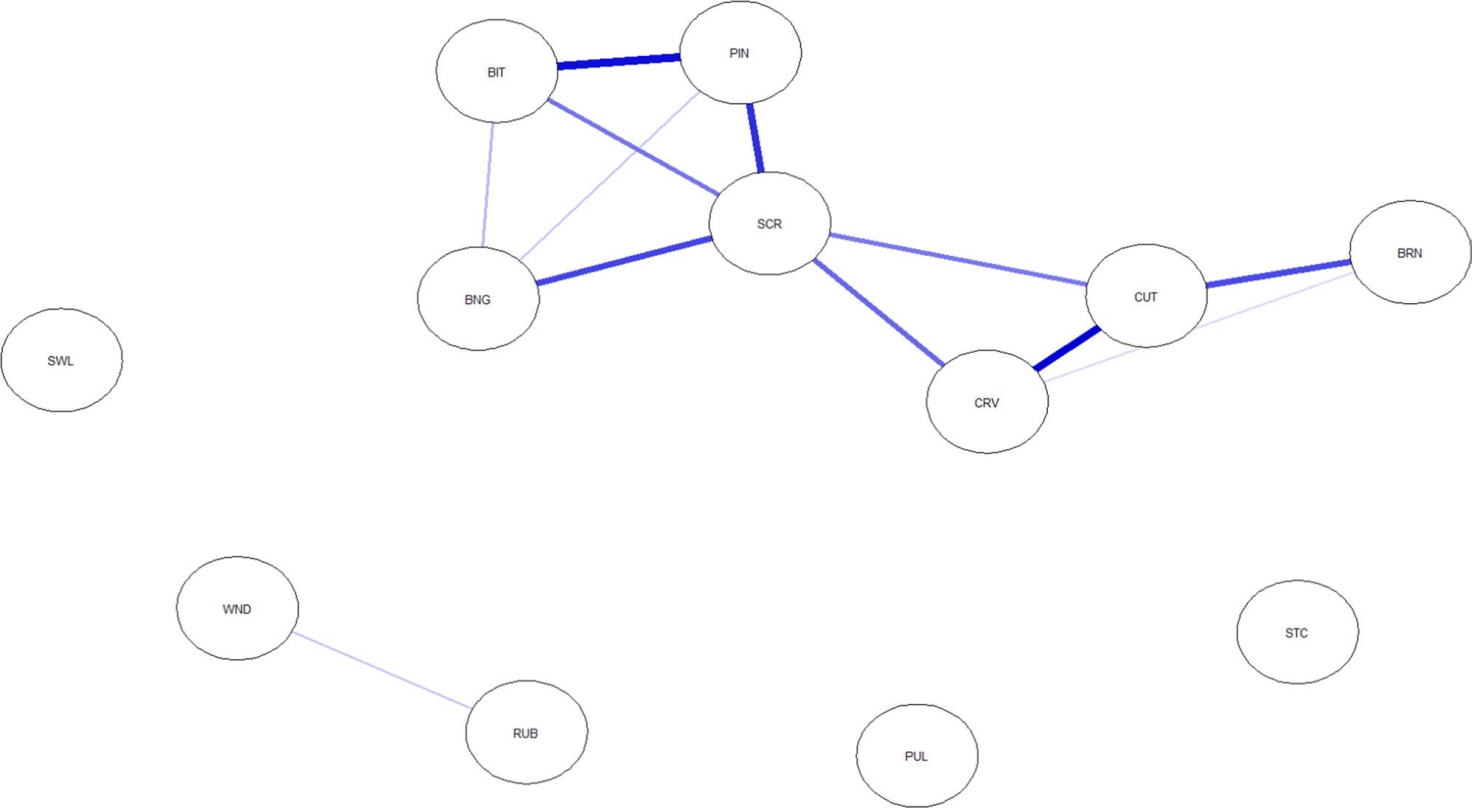
Network analysis of different nonsuicidal self-injurious (NSSI) behaviors Note. Blue edges represent positive associations. Thicker edges represent stronger relationships between NSSI behaviors. Node abbreviations are shown in Table 1

Figure 1 shows the network of NSSI behaviors. Edge weights from the network are presented in Supplementary Table 3. The network contained 54 zero edges (81.82%) and 12 positive edges (18.18%). The mean edge weights were 0.03 (range between 0.00 and 0.29) and 0.16 (range between 0.04 and 0.29) among all edges and among positive edges, respectively. The strongest positive edges (with a range of edge weights between 0.21 and 0.29) were between cutting and burning, between cutting and carving, between biting and pinching, between severe scratching and pinching, and between severe scratching and banging or hitting self.

#### Examining centrality of different NSSI behaviors

**Fig. 2:**
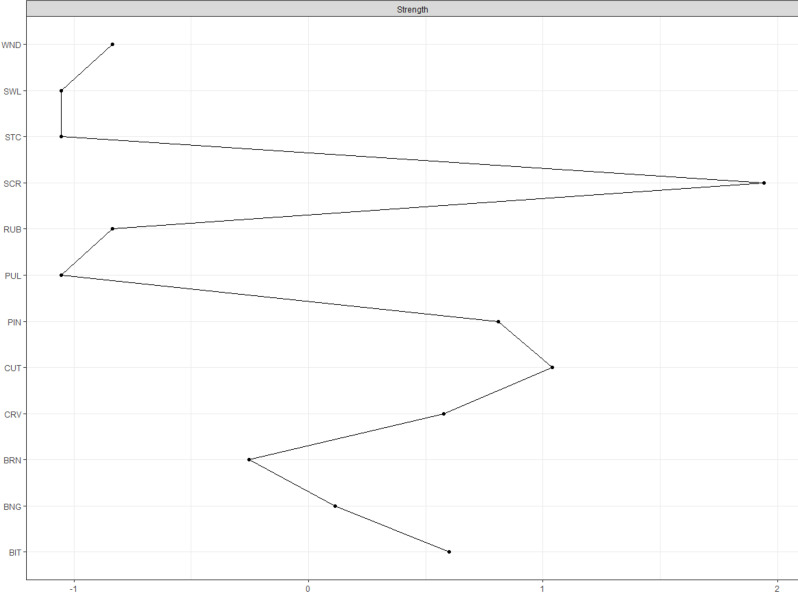
Standardized centrality index of strength related to the network of different nonsuicidal self-injurious (NSSI) behaviors *Note*. Node abbreviations are shown in Table 1

Figure 2 presents the standardized strength index for the network of NSSI behaviors. The NSSI behaviors with the highest strengths (ranging between 0.81 and 1.94) were severe scratching, cutting, and pinching. That is, these nodes had the highest rates of interrelationship with the other NSSI behaviors in the network (i.e., these nodes had more significant and higher assocations with other nodes). The lowest strength (with a value of -1.05) was presented for pulling hair, sticking self with needles, and swallowing dangerous substances.

Zhang’s clustering coefficients for the 12 NSSI behaviors are displayed in Supplementary Fig. 2. The highest levels on this index (ranging between 0.80 and 1.98) were presented for burning, banging or hitting self, and biting. Behaviors with high clustering coefficients might indicate the redundancy of these NSSI behaviors in the estimated network. The redundancy of NSSI behaviors can mean, for example, that limited additional information is provided by knowing the frequency of biting compared to other, more central NSSI behaviors.

#### Stability and accuracy of the network parameters

**Fig. 3:**
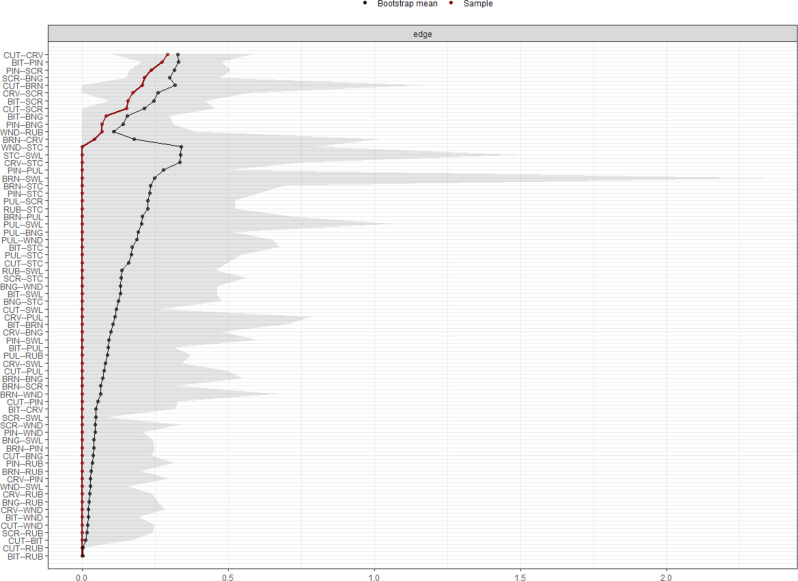
Accuracy of weighted edge estimates. *Note*. Weighted edge estimates from the sample (red dots and lines) are compared to weighted edge estimates from 1000 random bootstrap samples (black dots and lines) with confidence intervals (grayed area). Node abbreviations are shown in Table 1

Bootstrapped CIs for edge weights are shown in Fig. 3. Generally, relatively wide CIs were shown for each edge weight. Thus, the accuracy of the edge weights might be biased, and a cautious interpretation of the order of edge weights is warranted. However, the positive direction of multiple edge weights was supported (i.e., bootstrapped CIs did not cross 0). Compared to non-zero edge weights, those identified as zero in the sample-based network analysis showed greater deviation from the corresponding bootstrapped means of edge weights.

**Fig. 4:**
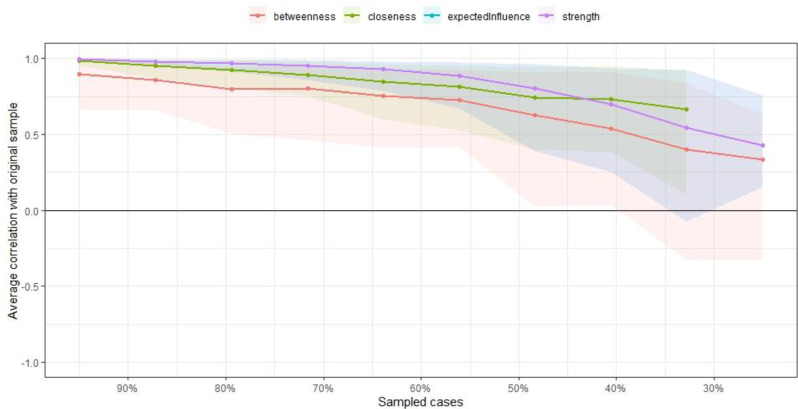
Correlation stability of the centrality index of strenghts between the original sample and subsets with increasing rates of dropped participants. *Note*. The solid lines indicate the means and colored areas indicate the range from the 2.5th quantile to the 97.5th quantile.

Figure 4 presents the findings of the case-dropping subset bootstrap analysis. The low stability of the strength index was indicated by a CS coefficient of 0.00, meaning that it was not possible to retain a correlation of at least 0.7 in at least 95% of the samples during bootstrap resampling. However, as the proportion of dropped participants increased from 0 to 50% (*N* = 707–417, respectively), the mean correlations between the original sample and subsets remained strong (*r* = 0.90–0.77) and ranged from moderate to strong levels in 95% of the samples (*r* = 0.36–1.00). A more marked decrease in correlation stability was observed when at least 60% of participants were dropped (*N* = 360 − 186). Overall, these findings suggest that the node-based order according to the strength index should be interpreted with caution, as this measure was not highly stable in the case-dropping samples.

**Fig. 5:**
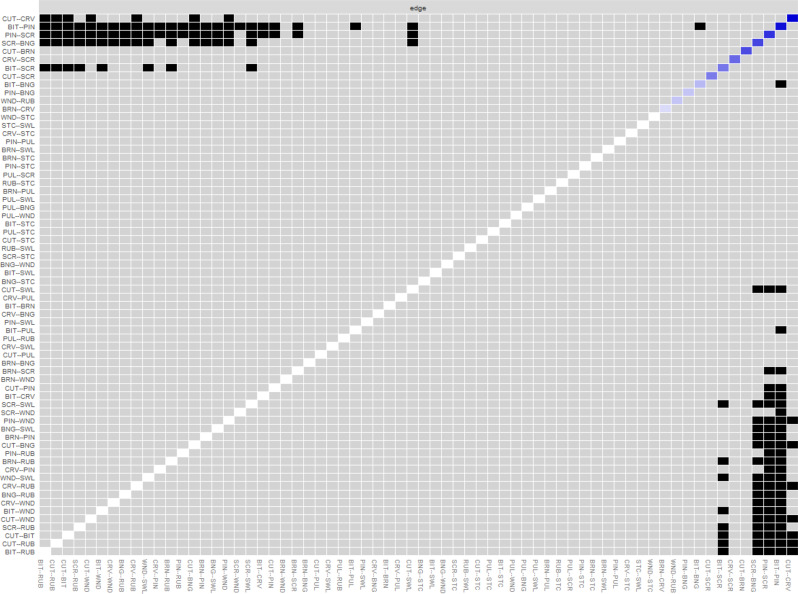
Bootstrapped difference tests for edge weights. *Note*. Grey boxes indicate non-significant differences, and black boxes show significant differences. Colored boxes in the diagonal indicate the strength of relationships. Node abbreviations are shown in Table 1

Figure 5 presents the results of bootstrapped difference tests for edge weights. The following edges were significantly (*p* < 0.05) stronger compared with multiple other edges: between cutting and carving, between biting and pinching, between severe scratching and pinching, between severe scratching and banging or hitting self, and between severe scratching and biting.

Supplementary Fig. 2 summarizes the results of bootstrapped difference tests for the centrality estimate of strength. Non-significant (*p* > 0.05) differences were observed for all comparison of NSSI methods in terms of strength. Consequently, the ranking of NSSI methods based on strength may be biased, warranting a cautious interpretation.

## Discussion

NSSI is often noted, particularly from a research perspective, as a perplexing phenomenon [34]. One reason for this may be the confusing diversity of the methods used. Therefore, we analyzed the interrelationship of 12 different self-injurious methods using network analysis based on a large community sample covering a wide range of adult ages. We have shown how the different NSSI methods are relatively positioned to each other in the network structure and how they interact.

Based on self-evaluation of the severity of 12 different NSSI methods, the majority of NSSI respondents reported using more than one form of self-injury. This trend is in line with Klonsky [22], who reported that half of adult self-injurers engaged in multiple methods [22]. In our study, the average number of NSSI methods engaged in was close to 4 (3.6 on average), or approximately twice the average number Klonsky [22] reported for a non-clinical adult self-injurious sample. However, the average found in our sample is consistent with a sample of first-year college students (M = 4.2; Wester et al. [47] and a sample of clinical adolescents (M = 3.7 among males, M = 4.0 among females; Nock et al. [33]. In contrast, the average number of NSSI methods was close to 6 in another study of clinically distressed adolescents [37]. Consistent with other studies using different methodologies, it seems to clearly be the case that a single NSSI method is rarely used in isolation, and instead, multiple methods are involved [6, 10, 21, 23], possibly because different NSSI methods interact or are linked in some way. The use of multiple methods is a serious problem because a growing number of studies also show that engagement in multiple NSSI methods is significantly associated with more psychopathological indicators than using a single method [39].

In our sample, the most commonly used NSSI methods were also similar to those reported in other community adult samples (e.g., Klonsky, [22]. Thus, hitting, interfering with wound healing, pinching, biting, and scratching skin are prominent among those who endorsed any form of NSSI. It is worth noting that the most common types of self-injury in the sample do not require any instrument (e.g., a sharp object) to perform them. They can be said to be “always at hand.” On the other hand, for the most part, these are low severity methods of NSSI [27].

Network analysis was used to assess the organizational structure of NSSI methods. In the variety of NSSI behaviors, patterns of interrelationship were identified. Although cautious interpretation is warranted due the low stability of the strength centrality index, the emerged NSSI network showed the highest centrality for cutting, severe scratching, and pinching. The first form is considered a more severe form of NSSI, can result in increased skin breakdown, and are more likely to be associated with bleeding. The strongest positive edges were between (1) biting, pinching, hitting self and severe scratching, and between (2) burning, skin carving and cutting. These more closely related methods can be said to form a group of NSSI methods. Because of the association present between the methods in a certain edge, we can assume that the NSSI acts linked in the edge occur more frequently together. For example, when one NSSI method appears, it may be that other related NSSI methods within the network are observed more frequently. But it is also hoped that if one method declines or disappears, the other associating behaviors will follow a similar downward trend. For the first edge, the four interconnected NSSI acts do not require any tools, whereas, for the second edge, an object (e.g., lighter, blade, needle) is required to cause self-injury. The linked NSSI methods in the second edge thus require more preparation and can cause more serious injury than the previous edge. This can be important for two reasons. On the one hand, limiting access to the means used for self-harm may be part of the treatment [26]. On the other hand, a detailed exploration of NSSI is part of assessing suicide risk. In addition to enduring NSSI and the absence of pain during NSSI, using multiple methods of NSSI is associated with an increased risk of suicidality [33]. Our study suggests that certain potentially co-occurring NSSI acts (e.g., skin carving, cutting, and burning) should be taken into account when estimating suicide risk. This also implies that the set of multiple methods of NSSI should be treated as a non-unitary entity and that it is worth looking for clusters within the larger set of methods. NSSI methods within a certain cluster may tend to act as interrelated entities, suggesting that each behavior could contribute to an increased likelihood of other behaviors occurring within the same cluster.

Severe scratching, pinching, and cutting played the most central role in the NSSI-methods network, i.e., have the strongest associations with other NSSI behaviors. Severe scratching and pinching have been among the most common NSSI-methods in other studies [5, 43] as well as in the present sample. In addition, cutting is associated with other NSSI modalities (i.e., burning and carving) that can cause more severe tissue damage, which can more easily result in the need for medical treatment. All of them are so-called impulsive self-harm acts, which usually result in superficial injury and are used by the person to bring immediate– albeit temporary– relief [32]. These acts can promptly reduce internal tension and painful emotional experiences by experiencing physical pain. In these cases, self-harm works as a way of emotion regulation. Cutting, which result in skin bleeding, can also be classified as more serious form of NSSI [27]. As hypothesized, this more severe form of NSSI has a more central role in the network of NSSI-methods. This result also supports the hypothesized pathway that more severe forms of NSSI strengthen the relationship between NSSI frequency and clinical severity [17].

Our findings, which highlighted a stronger association between certain forms of NSSI and its more significant impact on other forms of NSSI, can be incorporated into interventions and therapeutic techniques aimed at reducing self-harm (e.g., Motivational Interviewing; Cognitive Behavioural Therapy, Dialectical Behavioural Therapy, Problem-Solving Therapy). Each of these techniques emphasizes a deeper understanding of self-injurious acts. Detailed exploration of self-injurious episodes– which are most often built up from a specific series of self-injurious behaviors and thus have a specific history– is based on revealing of the circumstances and ways in which self-injury occurs. The NSSI methods identified as central in the network may be particularly important to pay attention to, espceially in the initial stages of treatment planning. At the beginning of treatment, it is worth using questionnaires that map several NSSI methods and their frequency in recent weeks. This process can also help to reveal the structure of the individual self-harm methods. Our research has pointed out that if this personal network includes severe scratching, cutting, and pinching, especially with increased episode rates, then reducing NSSI should be a major focus of treatment to minimize harm. However, it is important to emphasize that further studies in additional samples are needed and longitudinal evidence is essential before our results can be adopted in clinical settings.

Peripheral NSSI behaviors also appear in the network. Although cautious interpretation is warranted due the low stability of the strength centrality index, the emerged network showed the lowest centrality of pulling hair, sticking self with needles and swallowing dangerous substances. These are the three methods that have the lowest and fewest associations with other NSSI acts. According to the network model, pulling hair, sticking self with needles and swallowing dangerous substances are NSSI methods that are less related to, and fundamentally different from, other NSSI modes.

These most peripheral NSSI-methods represent the rarer forms of NSSI-frequency [49]. Hair pulling can be classified as a compulsive form of NSSI, as opposed to so-called impulsive methods (e.g., cutting, scratching, hitting self, burning; Møhl, [32]. Compulsive forms of NSSI are repetitive and often “ritualized” (e.g., hair-pulling may have a choreography that the person has developed, often involving eating the hair). Hair-pulling may not only be an NSSI behavior, but also a major symptom of trichotillomania syndrome (hair-pulling disorder), which belongs to the category of obsessive-compulsive or related disorders APA, [1].

Swallowing dangerous substances also had a peripheral role in the NSSI methods network. This form of self-injury raises questions. It is an ambiguous NSSI category due to its explicit severity and unclear danger limit, which is more closely related to suicidal self-harm [35]. Swallowing chemicals has low or the lowest lifetime prevalence as an NSSI method [49]. Thus, it is not surprising that in many classification studies, swallowing dangerous substances has not differentiated classes [38]. Further research might clarify whether these low centrality phenomena can indeed be included under the NSSI umbrella term [35].

The organizational structure of NSSI methods invites NSSI researchers and practitioners to consider several important questions. An important first question to address is whether our findings are replicable, particularly given that the accuracy and stability analyses did not support this. If so, other questions to consider include: Why are certain NSSI methods more central in the network? Which NSSI behavior pairs or interrelationship patterns are more typical? Are there important between-group differences in the interrelationships (e.g., in terms of gender, age, clinical vs. non-clinical groups)? What factors can affect the different edges of the network? What are the therapeutic implications of these network analysis results? Hopefully, findings from the current study will serve to facilitate further research in these related but different directions.

Although the present study is an innovative attempt to gain more information on the interconnectedness of seemingly separate NSSI methods, it also has several limitations. First, because of the cross-sectional design, it is not possible to infer causality or to detect trajectories of change in the NSSI network. On this basis, it would be worthwhile to conduct longitudinal studies that are able to map the interactions and developmental pathways between the different NSSI methods over time. Second, although the large community adult sample can be an advantage, we did not assess and therefore could not control diagnosis or history of mental illness due to ethical reasons. Third, although the sample covers a wide range of adult ages, the largest proportion of respondents fell into the under-40 category. This may explain the high NSSI lifetime prevalence rates in the overall sample, which is more typical among younger adult samples (cf., Wester et al. [47]. Another limitation was the imbalanced gender ratio in the total sample and in the NSSI sample. Consequently, gender differences were not tested. It is, therefore, important to evaluate the NSSI methods network in a more balanced adult sample in terms of age bands and gender ratios. A further aspect is that, although the interrelationships of several common NSSI methods were comprehensively investigated, not all existing NSSI methods (e.g., rubbing sandpaper on skin, dripping acid on skin, breaking bones) were covered. Furthermore, respondents completed the questionnaires online, which may have contributed to mainly younger adults in the sample and possibly people of average or higher socio-economic status participating. Both the recruitment and the informed consent indicated that NSSI would be included as a topic in the questionnaire package, which could have discouraged some respondents from participating. That is, the stigma associated with NSSI in society or even one’s involvement in NSSI may have affected the response rate and limited the external validity of our findings. In addition, the network parameters exhibited low stability and accuracy in the sensitivity analyses. Therefore, implications regarding the centrality of different NSSI methods and their associations should be drawn with great caution, as the present findings may have limited replicability. Finally, it should be acknowledged that the applied transformation of the original NSSI frequency data resulted in reduced variability and may have attenuated the richness of the original responses.

## Conclusion

The fact that NSSI is a heterogeneous phenomenon is already well established in the literature, but so far, little is known about the relationship between the heterogeneous NSSI methods. This gap exists because few studies have examined this interplay, and no studies have explored these associations using a network analysis approach. The current study applied network analysis to map and visualize the interconnections between a number of NSSI methods in a general adult sample. Severe scratching, cutting, and pinching emerged as the most central NSSI methods with the strongest and highest number of associations with the other NSSI behaviors in the network structure. This draws attention to the need to pay particular awareness to these NSSI methods in self-injury assessment because they may have a high probability of raising the risk of involvement in other NSSI behaviors. We found that it (i.e., cutting) is not necessarily the most frequent NSSI method that plays the most prominent role in the network of various NSSI acts and their interconnections. A more important aspect is versatility and the complex interplay of diversified NSSI behaviors. If a central NSSI method can be reduced or eliminated during psychological treatment, particularly in the case of cutting, severe scratching, and pinching, as suggested by this study, the use of related methods may be reduced, thus weakening the association between the frequency of NSSI and clinical severity.

## Electronic supplementary material

Below is the link to the electronic supplementary material.

Supplementary Material 1

## Data Availability

The dataset used and analysed during the current study is available from the corresponding author on reasonable request.

## Abbreviations

CIs: Confidence intervals
CS: Correlation stability
EBIC: Extended Bayesian Information Criterion
ISAS: Inventory of Statements About Self-Injury
LCA: Latent Class Analysis
LPA: Latent Profile Analysis
NSSI: Nonsuicidal self-injury
