# Supplementary material for: Interconnectivity among different nonsuicidal self-injurious methods – a network analysis
Source: BMC Psychiatry. 2025 Jul 1;25:601. doi: 10.1186/s12888-025-07045-2 (PMC12210547; doi:10.1186/s12888-025-07045-2)
Supplement: Supplementary file 1 — Supplementary Material 1 [file 12888_2025_7045_MOESM1_ESM.docx]

**Supplementary Materials**

**R Codes for the Analyses**

#Loading packages for the analyses

library(bootnet)

library(qgraph)

library(haven)

library(lavaan)

library(psych)

#Loading the dataset

nssidata <- read_spss("NSSI NWA.sav")

names(nssidata) <- c("CUT", "BIT", "BRN", "CRV", "PIN", "PUL", "SCR", "BNG", "WND", "RUB", "STC", "SWL")

#Estimating bivariate Spearman correlations

corr.test(nssidata, method = "spearman")

#Network analysis based on MGM

results <- estimateNetwork(nssidata,

default = c("mgm"),

labels = c("CUT", "BIT", "BRN", "CRV", "PIN", "PUL", "SCR", "BNG", "WND", "RUB", "STC", "SWL"),

type = c("c", "c", "c", "c", "c", "c", "c", "c", "c", "c", "c", "c"),

level = c(3, 3, 3, 3, 3, 3, 3, 3, 3, 3, 3, 3),

tuning = 0.5,

rule = "AND",

criterion = "EBIC",

threshold = "LW",

nFolds = 20)

#Weighted edges for the network

matrix <- print(results$graph)

#Plotting the network

plot(results)

#Centrality and clustering statistics for the network

centralityPlot(results,

scale = c("z-scores"),

include = c("Strength"),

labels = c("CUT", "BIT", "BRN", "CRV", "PIN", "PUL", "SCR", "BNG", "WND", "RUB", "STC", "SWL"))

clusteringPlot(results,

scale = c("z-scores"),

include = c("Zhang"),

labels = c("CUT", "BIT", "BRN", "CRV", "PIN", "PUL", "SCR", "BNG", "WND", "RUB", "STC", "SWL"))

#Bootstrapping analysis for the stability and accuracy of network parameters

boot1 <- bootnet(results, nCores = 8, nBoots = 1000, type = "nonparametric", statistics = c("edge", "strength"))

boot2 <- bootnet(results, nCores = 8, nBoots = 1000, type = "case", statistics = c("strength"))

#Plotting stability and accuracy analyses' results

plot(boot1,

order = "sample")

plot(boot1, "edge",

plot = "difference",

#onlyNonZero = TRUE,

order = "sample")

plot(boot1, "strength", order = "sample")

#Calculating correlation stability estimates

corStability(boot2)

**Supplementary Tables**

Supplementary Table 1. Descriptive statistics

| **Variable** | **N (valid cases)** | **Minimum** | **Maximum** | **Mean** | **Standard deviation** | **Variance** | **Skewness z-value** | **Kurtosis z-value** |
| --- | --- | --- | --- | --- | --- | --- | --- | --- |
| Cutting | 718 | 0 | >1000 | 8.94 | 65.13 | >1000 | 202.89 | >1000 |
| Biting | 713 | 0 | >1000 | 25.81 | 377.17 | >1000 | 289.56 | >1000 |
| Burning | 716 | 0 | 100 | 1.01 | 6.85 | 46.98 | 143.67 | >1000 |
| Carving | 715 | 0 | 100 | 2.26 | 8.79 | 77.33 | 86.67 | 407.56 |
| Pinching | 712 | 0 | 1000 | 19.03 | 83.79 | >1000 | 97.11 | 488 |
| Pulling hair | 711 | 0 | >1000 | >1000 | >1000 | >1000 | 296.22 | >1000 |
| Severe scratching | 715 | 0 | >1000 | 27.44 | 384.78 | >1000 | 274.44 | >1000 |
| Banging or hitting self | 709 | 0 | 1000 | 15.66 | 65.84 | >1000 | 123 | 829.39 |
| Interfering with wound healing | 707 | 0 | >1000 | >1000 | >1000 | >1000 | 295.44 | >1000 |
| Rubbing skin against rough surface | 713 | 0 | 1000 | 5.98 | 44.83 | >1000 | 185.44 | >1000 |
| Sticking self with needles | 712 | 0 | >1000 | 8.78 | 121.54 | >1000 | 244.22 | >1000 |
| Swallowing dangerous substances | 707 | 0 | 200 | 0.65 | 8.15 | 66.43 | 239.22 | >1000 |

Notes. To ease the interpretation of the descriptive statistics, values greater than 1000 are not reported with exact statistics. Skewness and kurtosis z-values are calculated by dividing the given statistical value with the corresponding standard error value.

Supplementary Table 2. Spearman’s bivariate Correlations Between the Study Variables

|  | **CUT** | **BIT** | **BRN** | **CRV** | **PIN** | **PUL** | **SCR** | **BNG** | **WND** | **RUB** | **STC** | **SWL** |
| --- | --- | --- | --- | --- | --- | --- | --- | --- | --- | --- | --- | --- |
| **CUT** | - | 0.04 | 0.25 | 0.31 | 0.01 | 0.05 | 0.22 | 0.11 | -0.02 | 0.06 | 0.22 | 0.12 |
| **BIT** |  | - | 0.16 | 0.08 | 0.35 | 0.17 | 0.33 | 0.26 | 0.03 | 0.06 | 0.10 | 0.08 |
| **BRN** |  |  | - | 0.16 | 0.10 | 0.21 | 0.18 | 0.17 | 0.12 | 0.16 | 0.26 | 0.21 |
| **CRV** |  |  |  | - | 0.06 | 0.06 | 0.27 | 0.15 | -0.01 | 0.14 | 0.24 | 0.08 |
| **PIN** |  |  |  |  | - | 0.27 | 0.36 | 0.25 | 0.11 | 0.09 | 0.18 | 0.07 |
| **PUL** |  |  |  |  |  | - | 0.22 | 0.18 | 0.18 | 0.19 | 0.20 | 0.15 |
| **SCR** |  |  |  |  |  |  | - | 0.29 | 0.03 | 0.09 | 0.10 | 0.07 |
| **BNG** |  |  |  |  |  |  |  | - | -0.01 | 0.12 | 0.17 | 0.07 |
| **WND** |  |  |  |  |  |  |  |  | - | 0.20 | 0.21 | 0.07 |
| **RUB** |  |  |  |  |  |  |  |  |  | - | 0.27 | 0.18 |
| **STC** |  |  |  |  |  |  |  |  |  |  | - | 0.14 |
| **SWL** |  |  |  |  |  |  |  |  |  |  |  | - |

*Note*. Node abbreviations are shown in Table 1.

Supplementary Table 3. Edge Weights of the Network Analyses

|  | **CUT** | **BIT** | **BRN** | **CRV** | **PIN** | **PUL** | **SCR** | **BNG** | **WND** | **RUB** | **STC** | **SWL** |
| --- | --- | --- | --- | --- | --- | --- | --- | --- | --- | --- | --- | --- |
| **CUT** | - | 0.00 | 0.21 | 0.29 | 0.00 | 0.00 | 0.15 | 0.00 | 0.00 | 0.00 | 0.00 | 0.00 |
| **BIT** |  | - | 0.00 | 0.00 | 0.27 | 0.00 | 0.16 | 0.08 | 0.00 | 0.00 | 0.00 | 0.00 |
| **BRN** |  |  | - | 0.04 | 0.00 | 0.00 | 0.00 | 0.00 | 0.00 | 0.00 | 0.00 | 0.00 |
| **CRV** |  |  |  | - | 0.00 | 0.00 | 0.17 | 0.00 | 0.00 | 0.00 | 0.00 | 0.00 |
| **PIN** |  |  |  |  | - | 0.00 | 0.24 | 0.07 | 0.00 | 0.00 | 0.00 | 0.00 |
| **PUL** |  |  |  |  |  | - | 0.00 | 0.00 | 0.00 | 0.00 | 0.00 | 0.00 |
| **SCR** |  |  |  |  |  |  | - | 0.21 | 0.00 | 0.00 | 0.00 | 0.00 |
| **BNG** |  |  |  |  |  |  |  | - | 0.00 | 0.00 | 0.00 | 0.00 |
| **WND** |  |  |  |  |  |  |  |  | - | 0.07 | 0.00 | 0.00 |
| **RUB** |  |  |  |  |  |  |  |  |  | - | 0.00 | 0.00 |
| **STC** |  |  |  |  |  |  |  |  |  |  | - | 0.00 |
| **SWL** |  |  |  |  |  |  |  |  |  |  |  | - |

*Note*. Node abbreviations are shown in Table 1.


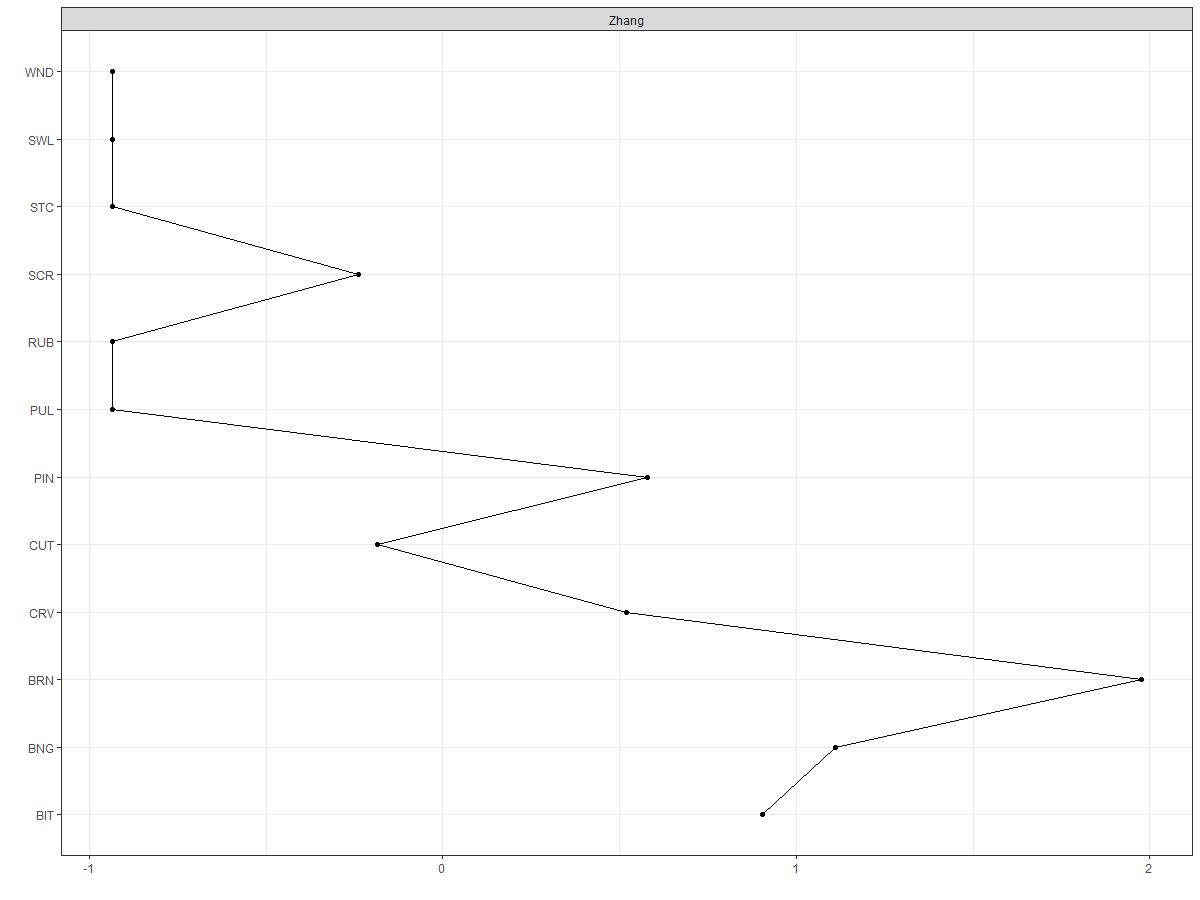


Supplementary Figure 1. Zhang’s standardized clustering coefficient related to the network of different nonsuicidal self-injurious (NSSI) behaviors. Notes. The y-axis presents different NSSI methods (node abbreviations are shown in Table 1), while the x-axis shows the standardized Zhang’s clustering coefficient for each method. Due to standardization, the mean clustering coefficient across all NSSI behaviors is 0, with a standard deviation of 1. A high (positive) Zhang’s clustering coefficient suggests that the given behavior has limited unique predictive value, as it exhibits substantial local connectivity and overlapping associations with related behaviors. The highest values were observed for burning (BRN), banging or hitting oneself (BNG), and biting (BIT). These high clustering coefficients may indicate redundancy of these behaviors within the estimated network. In contrast, a low (negative) clustering coefficient indicates that the behavior is weakly connected to its neighboring nodes, suggesting a more distinctive or independently functioning role. The lowest values were observed for interfering with wound healing (WND), swallowing dangerous substances (SWL), sticking oneself with needles (STC), rubbing the skin against a rough surface (RUB), and pulling hair (PUL). These latter NSSI methods may represent more distinct and independently functioning behaviors within the estimated network structure.


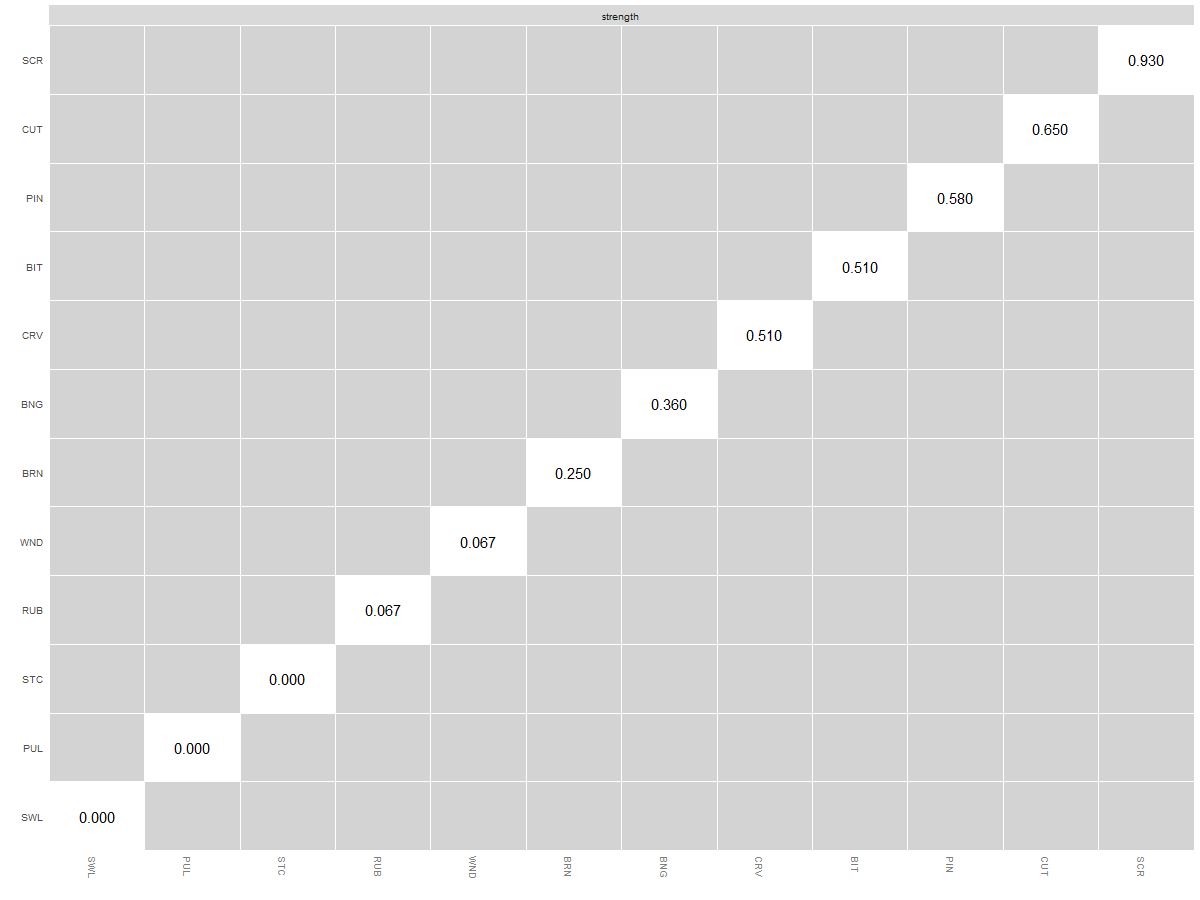


Supplementary Figure 2. Bootstrapped difference tests for the centrality index of strength. Notes. The analysis aimed to assess whether significant differences exist between the strength centrality values of individual NSSI behaviors. In the figure, behaviors are arranged along both axes in increasing order of strength; those positioned higher on the y-axis and further to the right on the x-axis reflect higher strength values. Node abbreviations are provided in Table 1. Values in the white diagonal boxes represent the raw (unstandardized) strength estimates for each node. Off-diagonal boxes display pairwise comparisons of strength centrality between different NSSI behaviors. These comparisons are redundant above and below the diagonal, as the results are symmetrical. Grey boxes indicate non-significant differences (*p* ≥ 0.05), and black boxes indicate significant differences (*p* < 0.05). No significant differences were observed between any pair of behaviors, suggesting that the apparent ranking of NSSI methods by strength (see Figure 2) should be interpreted with caution. For example, although severe scratching (SCR), cutting (CUT), and pinching (PIN) showed the highest strength values, these did not significantly differ from those of behaviors with the lowest strength values—pulling hair (PUL), sticking oneself with needles (STC), and swallowing dangerous substances (SWL).
